# Renal myopericytoma: A case report with a literature review

**DOI:** 10.3892/ol.2013.1678

**Published:** 2013-11-11

**Authors:** ZHIQIANG ZHANG, DEXIN YU, HAOQIANG SHI, DONGDONG XIE

**Affiliations:** Department of Urology, Second Affiliated Hospital of Anhui Medical University, Hefei, Anhui 230022, P.R. China

**Keywords:** myopericytoma, neoplasm, kidney, histopathology

## Abstract

Myopericytoma is a rare neoplasm that generally arises from the skin and superficial soft tissues of distal extremities, and is particularly rare in the visceral organs. The current report presents a case of giant myopericytoma showing kidney involvement, which is extremely rare. A 39-year-old male presented to the Department of Urology with a 2-month history of a painless and palpable mass in the region of the left abdomen. Unenhanced computed tomography revealed a 9×10×18-cm^3^ mass that was heterogeneous with central lower density. The patient underwent radical nephrectomy, including lymphadenectomy, without adjuvant therapy. The tumor was composed of spindle-shaped myoid cells with a concentric arrangement and showed immunoreactivity for smooth muscle actin and cluster of differentiation (CD)10, and had a Ki-67 index of <1%; however, staining was negative for CD34, desmin, S-100 protein, cytokeratin, human melanoma black (HMB)-45, B-cell lymphoma (Bcl)-2 and CD99. Routine follow-up revealed no local or distant metastatic signs of reccurrence for 20 months. The present report shows that renal myopericytoma may be a benign tumor, and surgical excision without adjuvant therapy may be the only potentially curative treatment approach.

## Introduction

Myopericytoma is a rare neoplasm that commonly arises from the skin and superficial soft tissues of distal extremities, and is particularly rare in the visceral organs (1). The present case of giant myopericytoma showing kidney involvement is an extremely rare occurrence. Myopericytoma demonstrates special morphological features composed of myoid-appearing oval or spindle-shaped cells with a concentric perivascular arrangement (1–3). In addition, myopericytoma exhibits immunoreactivity for muscle-specific and smooth muscle actin (1–4). The current report presents a case of renal myopericytoma, and a related literature review was performed to analyze the disease. Written informed consent was obtained from the patient.

## Case report

**Figure 1:**
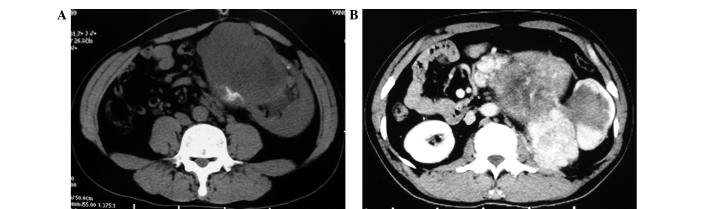
Tumor localized on the upper pole of the left kidney that is (A) heterogeneous with a central lower density and (B) showing heterogeneous attenuation with peripheral enhancement and central irregular non-enhancement.

**Figure 2:**
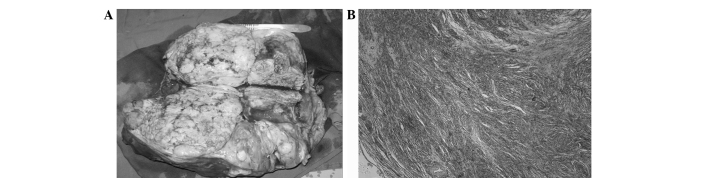
(A) Gross appearance and (B) histopathological examination of the resected specimen (hematoxylin and eosin; magnification, ×100).

**Figure 3:**
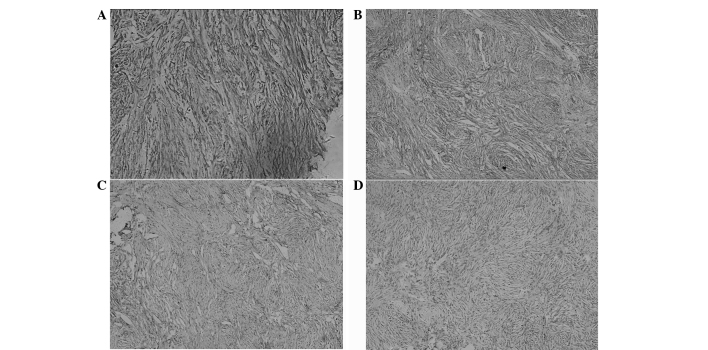
Immunohistochemically, the tumor cells were diffusely positive for (A) smooth muscle actin and (B) CD10, and (C) had a Ki-67 index of <1%, but were negative for (D) S-100 (magnification, ×100).

A 39-year-old male presented to the Department of Urology (Second Affiliated Hospital of Anhui Medical University, Hefei, China) with a 2-month history of a painless and palpable mass in the region of the left abdomen, and without a history of fever, weight loss, fatigue, urinary symptoms or hematuria. The patient presented with normal blood pressure and stable vital signs. Upon physical examination, no superficial lymph nodes were found. In addition, results from an electrocardiogram, pulmonary function test, stool analysis and other routine laboratory examinations were all within normal limits, with the exception of γ-glutamyltransferase (44 μmol/l). Unenhanced computed tomography (CT) revealed a 9×10×18-cm^3^ mass that was heterogeneous with a central lower density and showed a poorly defined margin with poor calcification. No invasion was identified of the ambient structures in the upper pole of the left kidney (Fig. 1A). Enhanced CT showed heterogeneous attenuation with peripheral enhancement and central irregular non-enhancement (Fig. 1B). However, no evidence of lung metastasis was found. The patient underwent radical nephrectomy, including lymphadenectomy, without adjuvant therapy. The gross appearance of the resected specimen of the giant mass showed a well-circumscribed, non-encapsulated, grayish-yellow solid tumor with areas of necrosis in black that measured ~20×13×10 cm^3^ (Fig. 2A). Histologically, the tumor was composed of spindle-shaped myoid cells with a concentric arrangement of cells around numerous variably-sized blood vessels, and the tumor cells were arranged in nests or fascicles (Fig. 2B). Nuclear atypia and mitotic figures were rarely found. Immunohistochemically, the tumor cells were diffusely positive for smooth muscle actin (Fig. 3A), cluster of differentiation (CD)10 (Fig. 3B) and had a Ki-67 index of <1% (Fig. 3C). However, staining was negative for CD34, desmin, S-100 protein (Fig. 3D), cytokeratin, human melanoma black (HMB)-45, B-cell lymphoma (Bcl)-2 and CD99. A routine follow-up demonstrated no signs of local or distant metastatic recurrence for 20 months.

## Discussion

Myopericytoma is a rare neoplasm that commonly arises from the skin and superficial soft tissues of the distal extremities, including the trunk, head and neck regions (1–4). In the majority of cases, myopericytoma is generally <4 cm in diameter, and its occurrence is particularly rare in the visceral organs. The present case of giant myopericytoma showing visceral organ involvement is extremely rare. A thorough review of previously published studies written in English revealed that renal myopericytoma was first reported by Lau *et al* in 2010 (1). The term myopericytoma was first proposed by Requena *et al*(3), and in 1998, Granter *et al*(2) specified the morphological and immunohistochemical characteristics of myopericytoma. In 2002, the World Health Organization bagan to use the term myopericytoma, and referred to it as a member of the pericytic group in the Classification of Tumors of Soft Tissue and Bone (5). Myopericytoma is morphologically heterogeneous and typified by oval/spindle-shaped cells with characteristic perivascular concentric growth and myoid differentiation (1–4). Immunohistochemical analysis of the tumor is positive for muscle-specific and smooth muscle actin, which are characteristic of myopericytoma and useful for its diagnosis and differential diagnosis (1–5). In addition, the tumor cells of myopericytoma have been found to express immunopositivity for desmin in a few cases. By contrast, in studies including the present case, immunohistochemical staining was negative for desmin, S-100 protein, cytokeratin and HMB-45 (1–5) Additionally, the present myopericytoma exhibited immunopositivity for CD10. The majority of myopericytoma cases, including the current case, are negative for CD34, a result which differs from that of another case previously reported in the literature (1).

Myopericytoma is generally considered a slow-growing neoplasm. Commonly, patients with renal myopericytoma are asymptomatic, with the tumor found incidentally by routine health checks. For this reason, an early diagnosis of myopericytoma is difficult for urologists. Ultrasonography, CT and MRI may highlight evidence of renal myopericytoma. Myopericytoma has atypical imaging features, although CT scans often show a heterogeneous density mass with peripheral contrast enhancement, unsmoothed margins and single or multiple slow-growing reactive lymph nodes (6).

The differential diagnosis of renal myopericytoma includes angioleiomyoma, glomus tumors, solitary fibrous tumors and myofibroma. Angiomyolipoma is the most common renal mesenchymal tumor, composed of variable thick-walled blood vessels, mature smooth muscle and mature fat. Angiomyolipoma is similar to myopericytoma in morphological features, and expresses immunoreactivity for HMB-45, S-100 and desmin, whereas myopericytoma rarely expresses immunoreactivity for desmin (7). Angiomyolipomas generally show a well-defined, circumscribed, hypodense mass on CT. The morphology and immunohistochemical features of myopericytoma are useful for its differential diagnosis. Glomus tumors exhibit a perivascular pattern of growth with cuboidal epithelioid cells, have an organoid pattern of the glomus organ and lack the characteristic perivascular concentric growth of myopericytoma (1,7–9). A solitary fibrous tumor is different from myopericytoma, as it exhibits immunoreactivity for the expression of vimentin, CD34, Bcl-2 and CD99 (1,10). In the present case, the absence of expression of CD34, CD99 and vimentin provided evidence for the differential diagnosis of renal myopericytoma. Myofibroma may exhibit a number of the characteristic microscopic features of mature bipolar myofibromatosis, including a zonal or biphasic architecture, fascicles of spindle cells and myoid nodules (1,8).

Although no standard treatment for renal myopericytoma has been established, complete surgical excision of the lesion may be the only potentially curative treatment. The clinical presentation and histological features of myopericytoma are usually benign, but a fraction of malignant myopericytomas with local recurrence or distant metastases have been reported. The size of the tumor does not necessarily correlate with malignant potential, but the distinction between benign and malignant variants has been determined by criteria with malignant features, including poor circumscription, high-mitotic activity, necrosis and nuclear pleomorphism (8,9). In the current case, the tumor appeared benign as the Ki-67 index was <1% and the mitotic activity was low; however, in contrast, it was >4 cm in size. A partial nephrectomy is performed for myopericytomas <4 cm in size, but larger tumors (>4 cm) may be treated by radical surgery. Chemotherapy or radiation therapy is unnecessary, although the timing and frequency of follow-up is essential. There is little available information with regard to targeted molecular therapies and prognosis; therefore, in the present case, the patient was treated with surgical exxision without adjuvant therapy.

In conclusion, renal myopericytoma is generally considered to be a relatively rare, slow-growing and benign tumor, with histological characteristics of the perivascular proliferation of myoid differentiated pericytic cells, which show a slow disease progression. Surgical excision may be the only potentially curative treatment for renal myopericytoma. However, the few previously reported cases may not be sufficient to allow the clinical outcome to be fully evaluated. Longer follow-up periods may also be necessary to definitively evaluate the clinical outcome of renal myopericytoma.
